# Meta-Analysis of the Safety and Efficacy of Direct Oral Anticoagulants for the Treatment of Left Ventricular Thrombus

**DOI:** 10.3390/ph17060708

**Published:** 2024-05-30

**Authors:** Mounica Vorla, Dinesh K. Kalra

**Affiliations:** 1 Department of Internal Medicine, Carle Foundation Hospital, Urbana, IL 61822, USA; mounica.vorla@gmail.com; 2 Division of Cardiology, University of Louisville, Louisville, KY 40292, USA

**Keywords:** anticoagulants, left ventricular thrombus, embolism, prescription, major adverse cardiac events

## Abstract

Background: Literature on the preferred anticoagulant for treating left ventricular thrombus (LVT) is lacking. Thus, our objective was to compare the efficacy of DOACs versus warfarin in treating LVT. Methods: Databases were searched for RCTs and adjusted observational studies that compared DOAC versus warfarin through March 2024. The primary efficacy outcomes of interest were LVT resolution, systemic embolism, composite of stroke, and TIA. The primary safety outcomes encompassed all-cause mortality and bleeding events. Results: Our meta-analysis including 31 studies demonstrated that DOAC use was associated with higher odds of thrombus resolution (OR: 1.08, 95% CI: 0.86–1.31, *p*: 0.46). A statistically significant reduction in the risk of stroke/TIA was observed in the DOAC group versus the warfarin group (OR: 0.65, 95% CI: 0.48–0.89, *p*: 0.007). Furthermore, statistically significant reduced risks of all-cause mortality (OR: 0.68, 95% CI: 0.47–0.98, *p*: 0.04) and bleeding events (OR: 0.70, 95% CI: 0.55–0.89, *p:* 0.004) were observed with DOAC use as compared to warfarin use. Conclusion: Compared to VKAs, DOACs are noninferior as the anticoagulant of choice for LVT treatment. However, further studies are warranted to confirm these findings.

## 1. Introduction

Left ventricular thrombus (LVT) is a dreaded complication in patients with myocardial infarction (MI) and dilated cardiomyopathy (DCM). Despite notable progress in managing these conditions, the occurrence of LVT persists at a considerable rate, varying between 4 and 39% in patients with acute MI [1] and 11–44% in those with DCM [2,3]. Depending on thrombus size and progression, LVT carries a risk of embolization of up to 22% [3,4,5,6] and a 37% risk of major adverse cardiovascular events (MACEs) [7].

To reduce the risk of thromboembolic (TE) events, clinical guidelines recommend anticoagulation for a duration of 3–6 months in patients with LVT. However, there seems to lack consensus among different societies regarding the choice of anticoagulation regimen. The 2013 American College of Cardiology/American Heart Association (ACC/AHA) ST segment elevation MI (STEMI) guideline recommends consideration of vitamin K antagonist (VKA) therapy for 3 months in patients with or at risk of LVT (e.g., those with anteroapical akinesis or dyskinesis) (Class IIb indication, level of evidence C) [8]. The 2023 European Society of Cardiology (ESC) guideline states that “the choice of (anticoagulant) therapy should be tailored to the patient’s clinical status and the results of follow-up investigations” but does not comment on the specific type of anticoagulant [9]. 

VKAs, predominantly warfarin, have been traditionally used for the prevention and treatment of LVT. However, difficulty in monitoring INR, drug–food and drug–drug interactions, and suboptimal times in therapeutic range (TTR) make warfarin a challenging therapeutic option for both providers and patients. Direct oral anticoagulant (DOAC) therapy, on the other hand, seems like an attractive option with fewer side effects while providing a more predictable and steady state of anticoagulation with enhanced patient compliance and fewer drug–drug interactions. Moreover, since inception, the cost of these drugs has fallen considerably. The 2022 AHA statement on the management of LVT indicates that DOAC therapy as a reasonable alternative to VKAs but does not comment on whether either anticoagulant is preferred [10]. In this context, our meta-analysis (meta-analysis) aimed to pool results from randomized clinical trials (RCTs) and observational studies to provide a more comprehensive understanding of the safety and efficacy of DOACs in LVT patients.

## 2. Methods

Our meta-analysis was conducted in accordance with the Preferred Reporting Items for Systematic Reviews and Meta-Analyses (PRISMA) 2020 guideline [11]. This study was registered with PROSPERO database (registration ID 550050) [12]. 

### 2.1. Data Sources and Searches

We conducted a literature search using the following Medical Subject Headings (MeSH) terms: “Direct Oral Anticoagulants”, “warfarin”, “Vitamin K antagonist”, and “Left ventricular thrombus”. PubMed, Cochrane, Google scholar, and ClinicalTrials.gov databases were systematically queried for all RCTs and observational studies comparing DOACs versus warfarin in patients with LVT and published between 1 January 1990 and 1 March 2024. Additionally, two investigators (MV and DK) independently reviewed the reference lists of identified studies and relevant reviews to identify additional pertinent studies.

### 2.2. Study Selection

Our meta-analysis encompassed all RCTs and adjusted observational studies comparing DOACs with warfarin in patients diagnosed with LVT. The following criteria were employed for study inclusion: confirmation of LVT diagnosis via cardiac imaging modalities such as transthoracic echocardiography (TTE) or cardiac magnetic resonance imaging (CMRi), a median follow-up period of at least 1 month, and the reporting of at least one clinical endpoint related to treatment approach. Excluded from our analysis were case reports, case series, cross-sectional studies, and single-arm investigations. Additionally, studies involving patients with intracardiac, ventricular mural, and right ventricular thrombus were excluded from our analysis.

### 2.3. Outcome Measures and Quality Assessment

The primary efficacy outcomes in our study included LVT resolution, systemic embolism, composite of stroke, and transient ischemic attack (TIA). Primary safety outcomes encompassed all-cause mortality and bleeding events. Additionally, major bleeding as defined by categories 3–5 according to The Bleeding Academic Research Consortium (BARC) [13] criteria or moderate–severe bleeding according to the Global Use of Streptokinase and t-PA for Occluded Coronary Arteries (GUSTO) criteria were also included in the safety outcome [13]. 

**Figure 1:**
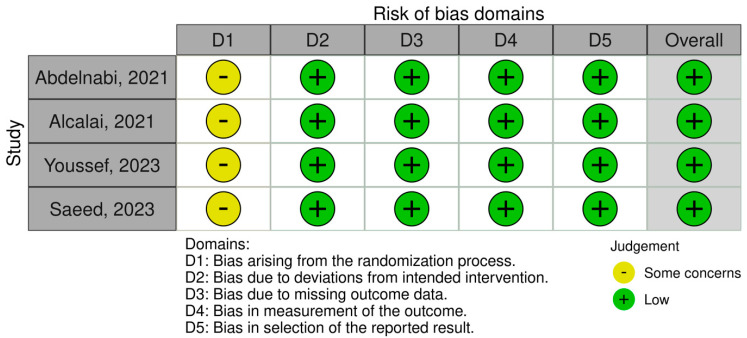
Assessing the risk of bias in randomized clinical trials.

To assess the quality of included observational studies and RCTs, we employed the Newcastle–Ottawa Scale (NOS) [14] and the Cochrane Collaboration Risk-of-Bias 2 (RoB 2) [15] tools. The NOS is a 9-point scoring system comprising f variables such as study selection, comparability of groups, ascertainment of exposure, and outcome measurement in observational studies, each allocated individual scores. Scores ranging from 0 to 3 indicate a very high risk of bias, 4 to 6 indicate a high risk of bias, and 7 to 9 indicate a low risk of bias (Table 1). On the other hand, the RoB 2 is a web-based tool developed in collaboration with Cochrane to assess the overall quality of RCTs based on variables such as randomization, deviation from intended intervention, outcome measurement, and selection of reported results (Figure 1). 

### 2.4. Data Synthesis and Statistical Analysis

Individual study-level data extraction was independently conducted by two reviewers (MV and DK) using a predefined form, which included information on study characteristics, baseline patient characteristics, and endpoint event rates.

Our meta-analysis was conducted according to the recommendations from Cochrane Collaboration using Review Manager, version 5.3 [43]. Pooled odds ratios (ORs) and 95% confidence intervals (CIs) were calculated using random-effects models with the Mantel–Haenszel method [44]. A *p*-value of less than 0.05 was deemed statistically significant for each clinical endpoint. The extent of heterogeneity among studies was assessed using the I^2^ statistic, with values exceeding 50% indicating significant heterogeneity. Forest plots were generated to visually depict the relative effect size of DOAC versus warfarin for individual clinical endpoints.

## 3. Results

**Figure 2:**
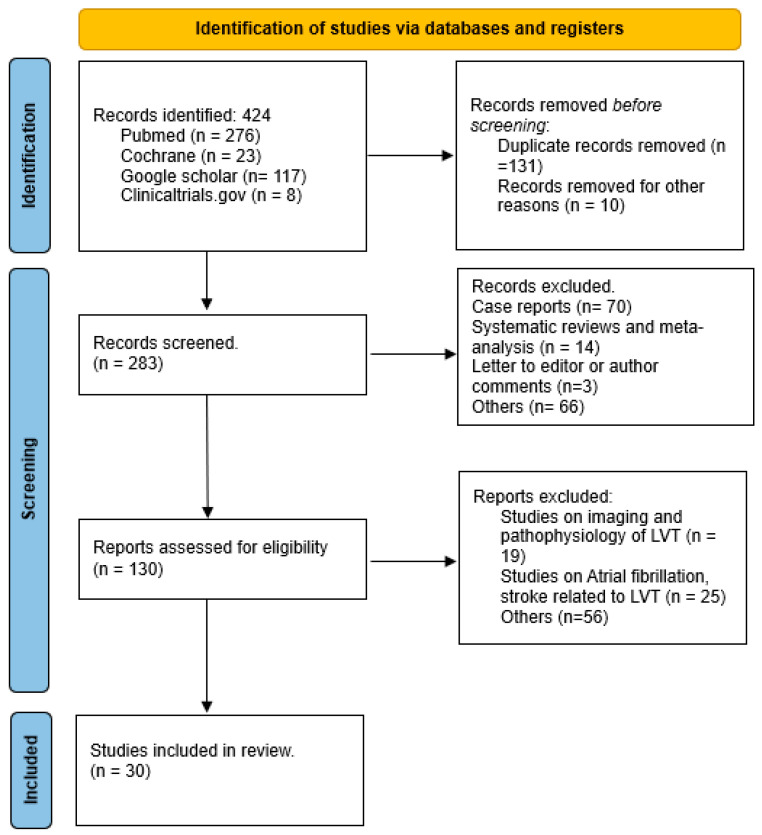
Preferred Reporting Items for Systematic Reviews and Meta-analyses flow sheet.

As depicted in Figure 2 the initial search yielded 424 publications. After reviewing titles and abstracts, 141 studies were excluded for lack of relevance. The remaining 283 articles underwent a comprehensive review and assessment to determine if they met the inclusion and exclusion criteria. Following a full-text review, 31 studies were included in the final analysis.

The included studies were homogeneous regarding the inclusion and exclusion criteria. Among these, 27 were observational studies, and 4 were RCTs [45,46,47,48]. Patients were followed for an average period of 16.9 months. The baseline characteristics of the patients in the included studies are summarized in Table 2. The mean age of the patients was 59 years. Of the study participants, 33% were treated with direct oral anticoagulants (DOACs) and 67% with warfarin. All studies included in the final analysis were deemed to have a low-to-intermediate risk of bias, as assessed using the Newcastle–Ottawa Scale (NOS) and Cochrane metrics for quality assessment.

### 3.1. Efficacy Outcomes

**Figure 3:**
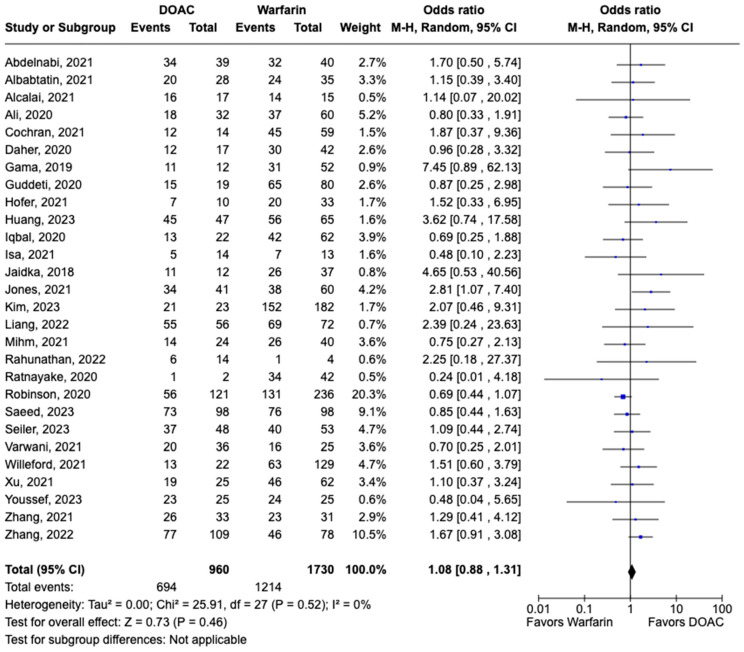
Forest plot of LVT resolution in trials, comparing DOAC vs. warfarin treatment groups.

**Figure 4:**
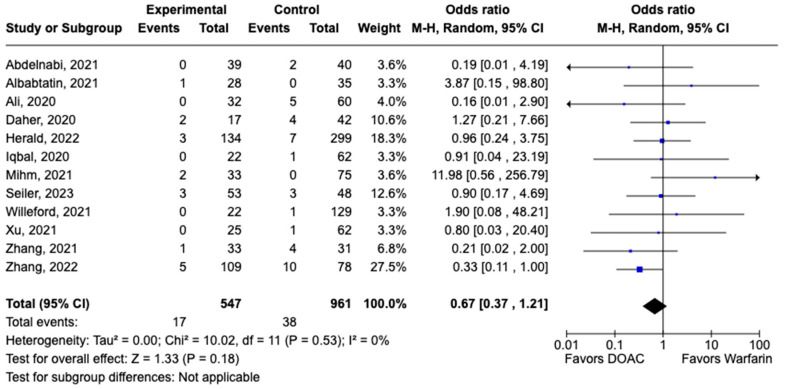
Forest plot of systemic embolism in trials comparing DOAC vs. warfarin treatment groups.

**Figure 5:**
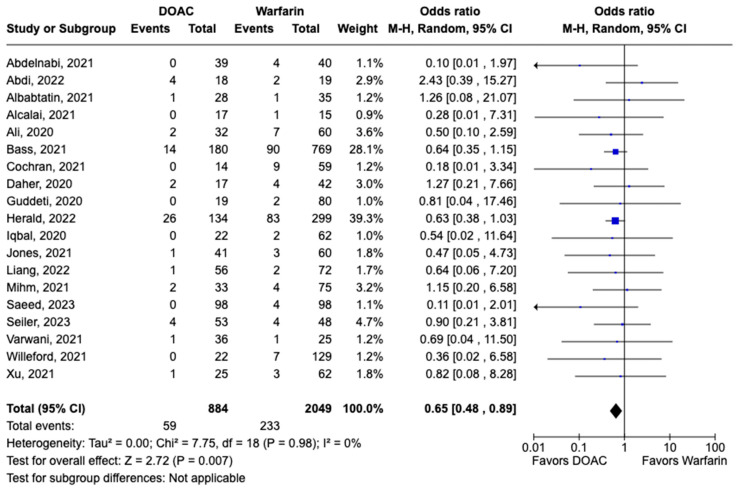
Forest plot comparing the occurrence of stroke/TIA in DOAC and warfarin groups.

LVT resolution was reported in 28 studies including 2690 patients. Compared with warfarin, DOAC use showed a trend toward higher odds of thrombus resolution (OR: 1.08, 95% CI: 0.86–1.31, *p:* 0.46) (Figure 3). The occurrence of systemic embolism was reported in 12 studies including 1508 participants. Although not statistically significant, DOAC use was associated with lowered risk of systemic embolism as compared to warfarin (OR: 0.67, 95% CI: 0.37–1.21, *p:* 0.18) (Figure 4). Additionally, 19 studies involving 2933 participants reported stroke/TIA. A statistically significant lower risk of stroke/TIA was observed in the DOAC group versus the warfarin group (OR: 0.65, 95% CI: 0.48–0.89, *p:* 0.007), with no heterogeneity (I^2^-0%) among the studies included in our analysis (Figure 5).

### 3.2. Safety Outcomes

**Figure 6:**
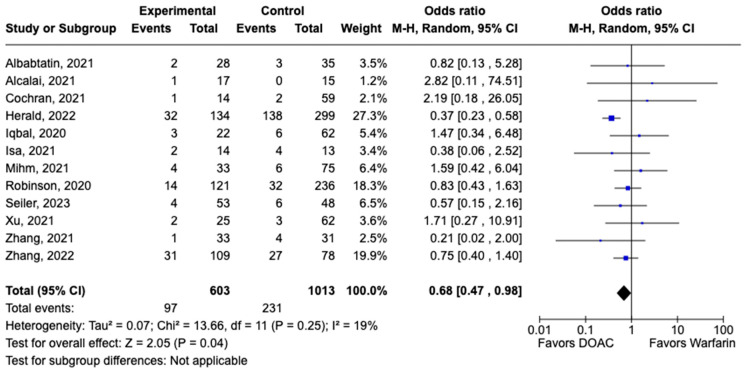
Forest plot comparing the occurrence of all-cause mortality in DOAC and warfarin groups.

**Figure 7:**
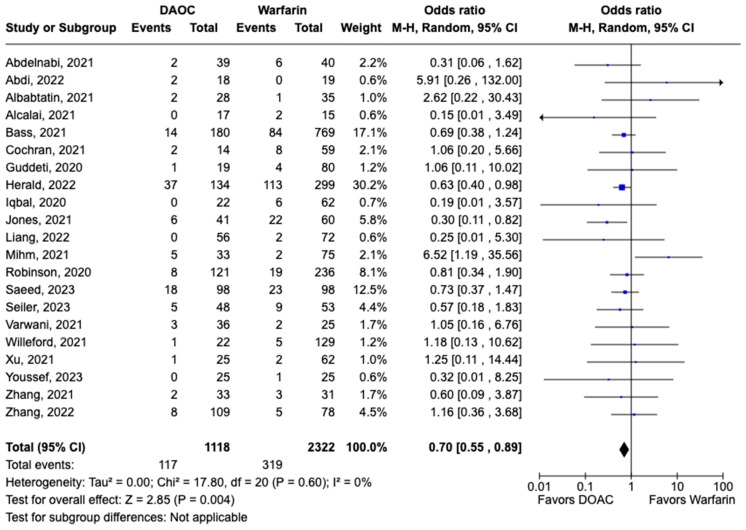
Forest plot comparing the occurrence of bleeding events in DOAC and warfarin groups.

**Figure 8:**
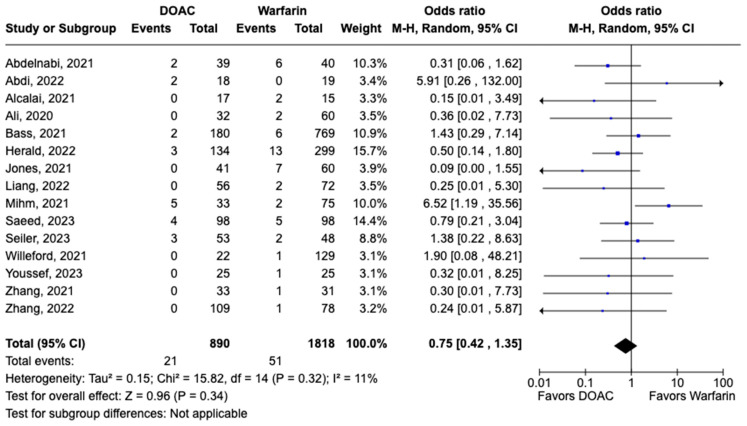
Forest plot comparing the occurrence of major bleeding in DOAC and warfarin groups.

All-cause mortality was reported in 12 studies including 1616 patients. There was a statistically significant reduced risk of all-cause mortality with DOAC use when compared with warfarin use (OR: 0.68, 95% CI: 0.47–0.98, *p:* 0.04), with mild heterogeneity (I^2^-19%) among the included studies (Figure 6). Bleeding events were reported in 21 studies including 3440 participants. DOAC use was associated with statistically significant lower odds of bleeding when compared with warfarin use (OR: 0.70, 95% CI: 0.55–0.89, *p:* 0.004), with no heterogeneity (I^2^-0%) among the studies included for analysis (Figure 7). Although not statistically significant, the risk of major bleeding was also lower in the DOAC group versus warfarin group (OR: 0.75, 95% CI: 0.42–1.35, *p:* 0.34) (Figure 8).

## 4. Discussion

LVT represents a concerning complication following acute MI, with an incidence of 3.5–8% [49,50,51] in the postpercutaneous coronary intervention PCI era. Likewise, incidences as high as 36–44% [2,3] and 68.5% [10] have been reported in anatomic pathology studies involving patients with DCM and heart failure (HF), respectively. Because of the heightened risk of TE complications, anticoagulation is imperative for preventing stroke and systemic embolism in patients with LVT. However, due to the scarcity of robust data, DOACs are merely recommended as alternatives to warfarin in patients with LVT requiring anticoagulation. Our meta-analysis including 31 studies is the most extensive comparison to date of DOACs vs. warfarin in patients with LVT. Random-effects analysis showed that DOACs are noninferior to warfarin for pharmacological anticoagulation in patients with LVT. In fact, DOAC use was associated with a significantly lowered risk of stroke/TIA, all-cause mortality, and bleeding when compared with warfarin use. 

Our meta-analysis results corroborate those of previous studies comparing DOACs vs. warfarin in patients with LVT. A subgroup analysis of seven studies [25,29,35,41,46,47] investigating the effect of DOACs with warfarin in patients after MI favored DOACs for LVT resolution (OR: 1.70, 95% CI: 0.94–3.07, *p:* 0.08). Similarly, two studies evaluated the effect of DOACs in patients with HF [42] and DCM [26]. The rates of LVT resolution were comparable between the groups but did not reach statistical significance. Given the distinct pathophysiological mechanisms in those after MI (including endocardial injury, inflammation, and blood stasis) and with HF/DCM (involving blood stasis, endothelial dysfunction, and hypercoagulability), further research exploring the impact of DOACs in different etiological contexts is warranted. Furthermore, the effect of concurrent antiplatelet therapy on LVT resolution and safety events needs investigation. 

The efficacy of rivaroxaban for LVT resolution has been evaluated in five studies [17,41,42,45,52]. Similarly, apixaban was assessed in three studies [28,46,47]. LVT resolution has occurred in 75% and 79% of patients treated with rivaroxaban and apixaban, respectively. Subgroup analysis favored rivaroxaban vs. warfarin; however, this difference did not reach statistical significance (OR: 1.26, 95% CI: 0.87–1.82, *p:* 0.23). Interestingly, the subgroup analysis of studies assessing apixaban for LVT resolution favored warfarin (OR: 0.55, 95% CI: 0.17–1.82, *p:* 0.33). The difference in outcomes between rivaroxaban and apixaban could be explained by different sample sizes and study design. Additionally, differences in thrombosis based on LVT etiology may reasonably translate into differences in anticoagulant responsiveness. Therefore, further research investigating the effect of different DOACs in patients with LVT is warranted. 

Finally, our study demonstrated that DOACs are no-inferior to warfarin as an anticoagulant of choice in patients with LVT. However, our study has a few limitations: (1) Most included studies are observational and nonblinded, raising concerns regarding missing data and selection bias. (2) With respect to meta-analyses, there is always the possibility of residual confounding and publication bias. (3) The imaging modality used to diagnose LVT (e.g., TTE vs. CMR) was not uniform across different studies. (4) There are no studies to date comparing the relative efficacy of different classes of DOACs (apixaban, rivaroxaban, dabigatran, etc.) with warfarin in patients with LVT. (5) We could not obtain data on adherence to DOACs or the time in the therapeutic range of warfarin treatment. (6) Finally, we were unable to standardize the dose of anticoagulants, but this also reflects the current dilemma of anticoagulation management in the LVT population in the real world. 

## 5. Conclusions

Since their introduction for treating venous TE and atrial fibrillation, DOACs have emerged as an appealing alternative to VKAs for both patients and clinicians. They offer advantages such as reduced need for monitoring, absence of dietary restrictions, and a lower risk of bleeding. Nevertheless, adequate data are lacking regarding the efficacy and safety of DOACs in managing LVT. Our meta-analysis demonstrates that that DOACs are comparable to warfarin in terms of efficacy (LVT resolution) and are associated with a decreased incidence of adverse events (bleeding). However, dedicated randomized clinical trials will be necessary to validate our findings and inform practice guidelines.

## Figures and Tables

**Table 1:** Assessing the risk of bias using Newcastle–Ottawa scale in observational studies.

	Selection				Comparability		Outcome			Overall Score
Study	Selection of Subjects Truly Representative/Not	Selection of Controls Drawn from the Same Cohort/Not	Ascertainment of Exposure Drawn from Secure Record/Self-Report	Demonstration of Outcome of Interest Absent/Present	Controlled for Baseline Characteristics Yes/No	Controlled for Other Factors Yes/No	Assessment of Outcome Drawn from Secure Record/Self-Report	Follow Up Length >3/<3 Months	Adequacy of Follow-Up <20%/>80% Lost to Follow-Up	
Abdi, 2022 [16]	1	1	1	1	1	0	1	1	0, 30% lost to follow-up	Good
Albabtain, 2021 [17]	1	1	1	1	1	0	1	1	1	Good
Ali, 2020 [18]	1	1	1	1	1	1	1	1	1	Good
Bass, 2022 [19]	1	1	1	1	1	0	1	NR	1	Good
Cochran, 2021 [20]	1	1	1	1	1	0	1	1	1	Good
Daher, 2020 [21]	1	1	1	0	0	0	1	NR	1	Fair
Gama, 2019 [22]	1	1	1	1	1	1	1	NR	1	Good
Guddeti, 2020 [23]	1	1	1	0	1	1	1	1	1	Good
Herald, 2022 [24]	1	1	1	1	1	1	1	1	1	Good
Hofer, 2021 [25]	1	1	1	1	1	0	1	1	1	Good
Huang, 2023 [26]	0, included only DCM patients	1	1	1	1	1	1	1	1	Good
Iqbal, 2020 [27]	1	1	1	1	1	1	1	1	1	Good
Isa, 2020 [28]	1	1	1	1	1	1	1	1	1	Good
Jaidka, 2018 [29]	0, only AMI patients	1	1	1	1	1	1	1	0	Good
Jones, 2021 [30]	0, only AMI patients	1	1	1	1	1	1	1	1	Good
Kim, 2023 [31]	1	1	1	1	1	1	1	1	1	Good
Liang, 2022 [32]	0, only AMI patients	1	1	1	1	1	1	1	1	Good
Mihm, 2021 [33]	1	1	1	1	1	1	1	1	0	Good
Rahunathan, 2023 [34]	1	1	1	1	1	1	1	1	1	Good
Ratnayake, 2020 [35]	0, only AMI patients	1	1	1	0	0	1	1	1	Fair
Robinson, 2020 [36]	1	1	1	1	1	1	1	1	1	Good
Seiler, 2023 [37]	1	1	1	1	1	1	1	1	1	Good
Varwani, 2021 [38]	1	1	1	1	1	1	1	1	1	Good
Willeford, 2021 [39]	1	1	1	1	1	1	1	1	1	Good
Xu, 2021 [40]	1	1	1	1	1	1	1	1	1	Good
Zhang, 2021 [41]	0, only AMI patients	1	1	1	1	1	1	1	1	Good
Zhang, 2022 [42]	0, only HF patients	1	1	1	1	1	1	1	1	Good

NR—not reported.

**Table 2:** Baseline characteristics of studies included.

Author, Year	Type of Study	Total Participants, n	DOAC/VKA Group, n	DOAC/VKA Group Mean Age, Years	Women, n (DOAC/Warfarin)	CAD, % (DOAC/Warfarin)	CHF, % (DOAC/Warfarin)
Abdelnabi, 2021 [45]	RCT	79	39/40	NR	NR	NR	NR
Abdi, 2022 [16]	Observational	40	18/19	NR	NR	NR	NR
Albabtain, 2021 [17]	Observational	63	28/35	58/59	4/1	NR	NR
Alcalai, 2021 [46]	RCT	35	18/17	56/59	5/2	18/22	NR
Ali, 2020 [18]	Observational	92	32/60	59/58	6/11	NR	78/75
Bass, 2022 [19]	Observational	949	180/769	63/62	55/224	43/57	68/75
Cochran, 2021 [20]	Observational	73	14/59	52/62	3/14	53/61	73/81
Daher, 2020 [21]	Observational	59	17/42	57/61	3/7	88/74	NR
Gama, 2019 [22]	Observational	66	13/53	69/69	NR	NR	NR
Guddeti, 2020 [23]	Observational	99	19/80	61/61	4/25	58/66	100/96
Herald, 2022 [24]	Observational	433	134/299	66/65	18/57	35/36	88/88
Hofer, 2021 [25]	Observational	43	10/33	NR	NR	NR	NR
Huang, 2023 [26]	Observational	122	47/65	49/39	9/12	NR	NR
Iqbal, 2020 [27]	Observational	84	22/62	62/62	2/7	NR	95/94
Isa, 2020 [28]	RCT	27	14/13	55/55	1/1	NR	NR
Jaidka, 2018 [29]	Observational	49	12/37	57/61	3/9	0/8	NR
Jones, 2021 [30]	Observational	111	41/60	59/67	7/9	NR	NR
Kim, 2023 [31]	Observational	205	23/182	NR	NR	NR	NR
Liang, 2022 [32]	Observational	128	56/72	55/55	5/10	NR	NR
Mihm, 2021 [33]	Observational	108	33/75	63/60	7/9	NR	NR
Rahunathan, 2023 [34]	Observational	18	14/4	59/64	2/1	NR	NR
Ratnayake, 2020 [35]	Observational	44	2/42	NR	NR	NR	NR
Robinson, 2020 [36]	Observational	357	121/236	58/58	27/66	NR	NR
Saeed, 2023 [48]	Observational	196	98/98	56/56	17/22	NR	13/11
Seiler, 2023 [37]	Observational	101	48/54	64/62	6/12	NR	6/10
Varwani, 2021 [38]	Observational	92	58/34	NR	NR	NR	NR
Willeford, 2021 [39]	Observational	151	22/129	54/56	5/25	NR	86/85
Xu, 2021 [40]	Observational	87	25/62	59/62	6/15	NR	NR
Youssef, 2023 [47]	RCT	100	25/25	52/54	NR	NR	NR
Zhang, 2021 [41]	Observational	64	33/31	60/61	9/8	NR	NR
Zhang, 2022 [42]	Observational	187	109/78	65/63	24/12	97/61	59/39

NR—not reported.

## Abbreviations

ACC	American College of Cardiology
AHA	American Heart Association
BARC	Bleeding Academic Research Consortium
CI	Confidence interval
CMRi	Cardiac magnetic resonance imaging
DCM	Dilated cardiomyopathy
DOAC	Direct oral anticoagulant
ESC	European Society of Cardiology
GUSTO	Global Use of Streptokinase and t-PA for Occluded Coronary Arteries
HF	Heart failure
LVT	Left ventricular thrombus
meta-analysis	Meta-analysis
MACE	Major adverse cardiovascular events
MeSH	Medical Subject Headings
MI	Myocardial infarction
NOS	Newcastle–Ottawa Scale
OR	Odds ratio
PRISMA	Preferred Reporting Items for Systematic Reviews and Meta-Analyses
RCT	Randomized clinical trial
RoB 2	Risk-of-Bias 2
STEMI	ST segment elevation myocardial infarction
TE	Thromboembolic events
TIA	Transient ischemic attack
TTE	Transthoracic echocardiography
TTR	Time to achieve therapeutic range
VKA	Vitamin K antagonist
